# Does Sociology Have Any Choice but to Be Evolutionary?

**DOI:** 10.3389/fsoc.2019.00006

**Published:** 2019-02-26

**Authors:** Edmund Chattoe-Brown

**Affiliations:** School of Media, Communication and Sociology, University of Leicester, Leicester, United Kingdom

**Keywords:** evolutionary sociology, functionalism, selectionism, organizational ecology, agent-based modeling, genuine novelty, historical change, rationality

## Abstract

Historically, over the long run, evolutionary approaches have struggled in sociology with great effort being expended (sometimes purely rhetorically rather than scientifically) to criticize them or, even more radically, to rule them out of court altogether as “not sociological.” This approach implies that such approaches are optional to the sociological project. By contrast, this article takes an opposing position and argues that sociology has no real alternative to evolutionary approaches in at least two key areas. First and foremost, we need an approach that can explain social organization without relying on implausible levels of deliberation (while still compatible with the, sometimes successful, exercise of reason). Secondly, we need an approach that is “properly” historical in being able to engage with both macro (structural) change and genuine novelty. This article not only discusses what is needed and why but also illustrates how such an approach could work using an Agent-Based Model (hereafter ABM).

## Introduction

Evolutionary analysis (as broadly construed as possible—see Dickens, 2000 for a useful if conceptually incomplete discussion) has a checkered history in sociology. Social Darwinism is now deeply disapproved of both intellectually and ideologically (Hodgson, 2004), as are the reductionist claims of sociobiology (Wilson, 1975). In many quarters functionalism is believed to be completely discredited and has moved beyond that to not even being considered worth teaching. The latest discussion I can find in a textbook is from Lee and Newby (1994). Nonetheless, there remain a number of ideas such as selectionism (Runciman, 1998), organizational ecology [for example (Hannan and Freeman, 1988)] and neofunctionalism (Alexander, 1998) that suggest that evolutionary ideas have not completely died out in sociology^1^.

This article takes what is, I hope, a novel approach to this long-standing debate. Instead of arguing, as many have done before, about the pros and cons of evolutionary sociology in general or specific evolutionary approaches in particular (Chattoe, 2002), it considers whether sociology, seeking to explain what it apparently seeks to explain, can really afford *not* to acknowledge evolutionary accounts (whatever criticisms may be leveled at the existing contenders for this role). Thus instead of asking what the evolutionary accounts *are*, I ask what evolutionary accounts can *do for* sociology that cannot be done in any other way. Of course, by its nature, such a project cannot be strictly objective. Sociologists, perhaps more than any other group of scholars, disagree about what their own subject is and how it should be done. But I hope to show (by the time my argument is completed) that a sociology that decides to disallow evolutionary accounts completely is not one that many sociologists would in fact be happy with. (It can be seen as a testament to the widespread dislike of evolutionary accounts in sociology that the field has not apparently considered how much it is obliged to give up by taking this position).

## The Structure of the Argument

It is possible to approach the argument from two different perspectives which we might loosely refer to as the “theoretical” and the “empirical.” By the theoretical I mean all those generalized explanations that have been offered for social phenomena from very micro/interactional accounts (like rational choice or social construction) to very macro ones (like the Marxist theory of historical change as class struggle). By the empirical (which, of course, can both be informed by and inform the theoretical) I mean the commonplace research that dominates the sociological literature when non-empirical theoretical debate is removed (regression analyses of survey data, justified narrative accounts based on qualitative interviews and so on).

Unusually, it is easier to start the argument with the theoretical element. The fundamental point here is that, regardless of the degree of individual “rationality” presumed by different kinds of theories, all the way from “strong” rational choice to Marxist false consciousness, unless we assume that it is possible for social actors to have perfect “models of the world” (and “perfect” models would seem to imply, for example, that predictions based on those models were always correct which gives us pause in credibly arguing that we might assume this) then the system inevitably involves unintended consequences and it is these unintended consequences which then have the capability to act differentially on those with different resources or views of the world thus potentially “selecting” some kinds of social actors (or groups or organizations) at the expense of others. If we recognize the implications of this point (that perfect models are implausible and that without them we need to address the issue of unintended consequences), then we also need a tool capable of modeling these selection effects. As I show in the middle of this article, ABM is potentially such a tool.

This insight is already somewhat at variance with the prevailing sociological perspective. Generally, sociology does not think in terms of “models of the world” to begin with and, for example, it is really hard to articulate a distinctively sociological theory of decision and choice (except to the extent that sociology does *not* predominantly wish to use the insights developed by economics and psychology). However, all that is required for the argument at present is something that most sociologists would (I imagine) be obliged to accept, namely that social actors *do* make choices, at least sometimes, and that these choices have something to do with the real world [although the argument allows that this connection may be quite weak—as when, for example, social actors take steps based on a belief in impending apocalypse even when all the previous predictions of their religious group in this regard have been falsified—(Festinger et al., 1956)]. In the next section, I shall present a simple example that makes it much easier to see how selection based on unintended consequences would operate. This is a preamble to using the general idea in a specific ABM.

## A Case Study: Alchian and Evolutionary Firm Behavior

Interestingly, the argument about selection of imperfect models was put forward early and particularly clearly by an economist, Armen Alchian (Alchian, 1950) for the case of firm behavior^2^. This example is worth discussing because it is clear and intuitive, allowing access to the reasons why unintended consequences are almost certain (or, put another way, why perfect models of the world are probably impossible). One of the many things that firms have to do is set a price for their product. This price will certainly be constrained at a lower bound by the costs of production (though the firm will also be constantly exploring ways to reduce these). It may be further constrained by the practices of the firm itself (for example insisting on a 10% profit margin in order to continue operating in a particular industry). It is also very likely, for the purposes of argument, that the firm will be highly “intelligent” (though not necessarily “rational” in the formal sense) in finding out as much as it can about the state of the market (what prices others are charging, what production processes they are using that give some insight into their likely costs and therefore the feasibility of undercutting them and so on)^3^. But at the end of the day, whatever a particular firm decides to charge in order to survive (and perhaps thrive) in the market can only be an intelligent hypothesis not an infallible prediction precisely because a market consists of autonomous social actors.^4^ For example, a firm may decide to cut its price so it makes almost no profit, hoping to gain market share, only to find that another firm has cut its prices even lower, based on having secretly retained profits, specifically aiming to drive out competition. This strategy probably could not be inferred from anything the first firm could observe, precisely because the second firm would be taking all possible steps to keep it secret (since it might actually be illegal). In this changed circumstance, the first firm could easily be the first to go to the wall (even if it attempted to put its prices back up) because it was *neither* offering a competitive price as it intended *nor* achieving any profit to keep it afloat^5^.

This example is also useful because it illustrates some of the potential pitfalls of existing informal evolutionary accounts in sociology. A firm is a legal entity and it is therefore *relatively* straightforward to say when it comes into being and ceases to exist (along with who works in it)^6^. It also relates formally to other entities in society in ways that define its survival possibilities. (Although a firm may borrow money for periods and perhaps delay paying its suppliers to ease a cash flow problem, in the long term it *has* to keep covering its production costs—which may include, for example repayment on loans—with sales revenues to persist. If it *doesn't* have to do this, then it isn't really a firm in the accepted sense but a state enterprise or a rich person's hobby). This account can be compared with the famous Parsonian example of the nuclear family (Parsons, 1956). A nuclear family is *not* predominantly a legal entity and it is unclear what it means for a family to cease to exist (unless family members actually die). People can live apart, not like each other very much and yet still consider themselves “family” (as well as doing the sort of things that families do like supporting each other in a crisis). This point does not undermine the plausibility of Parsonian claims that certain family structures may be better at reproducing themselves (and their advantages) than others [(Bourdieu and Passeron, 1990) but also see (Sullivan, 2001)] but it does problematize the idea that this phenomenon can coherently be represented as a selection process. Family structures are not “in competition” for a finite resource in the straightforward way that firms are^7^. Thus any evolutionary analysis has to be careful to present a coherent account of exactly what is being selected and by what. This requires a certain amount of formality in the specification (which an ABM, as illustrated later in the article, can and does provide)^8^.

To recap then, in the long term, firms must make a profit (sales revenues must exceed production costs by however little). To do this they have a number of decisions at their disposal based on a developing “model of the world” (how to produce, what price to charge, how much to spend on advertising and so on). But at the end of the day, no matter how carefully reasoned and evidentially based these decisions are, it is the “market” that will decide the actual outcome (based in particular on the joint decisions of *other* firms which may be deliberately obscured). And this outcome may be worse for firms that have made certain decisions (or have particular features like being poor, or new, or keeping little inventory) than for others. Thus, over time, some strategies or kinds of firms will persist in the market and others will disappear^9^. This is a classic example of a social evolutionary process. As we shall see later in the article, the same reasoning can be applied to a social (rather than economic) context. Agents displaying some genetic, cultural or social patterns of behavior will survive and reproduce at the expense of others as a result of “the environment.” As in the case of firm competition, the kind of “rationality” required to anticipate all the actions of other agents in such a system and thus take an action subject to no unintended consequences is probably unachievable.

There are other reasons too why we should not expect social actors to be able to develop perfect models of the world apart from independent joint action and genuine uncertainty: Will the Brazilian coffee crop be destroyed by frost next year? So far we have treated “the market” as an objective and unchanging thing but one side effect of, for example, its perceived injustices may be that the government decides (either exogenously or under political pressure) to change the terms on which firms may trade. Or firms may themselves, either legally or illegally, attempt to alter the structure of the market in their favor: A big firm may be able to undercut and drive out competition or lobby for changes in the law that particularly suit its operation (see the discussion of Enron by Wheen, 2004, pp. 276-285). Thus the nature of the environment means that although firms may have very good models of the market and be taking all sensible steps to use and improve them, a considerable capacity for unintended consequences is likely to remain. (To take an extreme example, consider the collapse of Barings Bank, allegedly the result of the actions of a single “rogue trader,” Nick Leeson.) In the model presented shortly, where the action studied is foraging for food, the actions of every agent affect (via the state of the environment) the survival possibilities for every other agent^10^.

Thus, unless sociologists are prepared to countenance the possibility of perfect “models of the world” held by individuals, it seems that they must recognize (and attempt to engage with) the possible selective role of unintended consequences in social systems and use tools that allow them to represent and analyse systems with those properties.

## From Theory to Data: Evolution as a Historical Process

This point, and particularly the observation that evolutionary processes necessarily occur over time now allows us to link the argument to the empirical dimension. Although general arguments about whether sociology needs to be “more historical” are probably as futile as those about whether it needs to be “more evolutionary,” specific arguments can be put forward that suggest why evolutionary accounts may be necessary to a coherent and believable sociology and some of these arguments conveniently introduce the empirical strand of the issue.

Consider a regression analysis of cross sectional survey data. Regardless of any reservations about the ability of regression to give insight into causation and explanation, it defines a relationship in the data and gives a rigorous and clear sense of how “good” that relationship is. Now suppose that it was possible to carry out the same analysis on comparable data collected a decade before^11^. If the parameters were “the same” (whatever that is assumed to mean in practice) would we conclude that nothing important had changed in the decade?^12^ What would we conclude (or could we legitimately conclude) if the parameters had changed significantly? Even leaving aside the impact of history with a capital H, sociology cannot avoid the problem of change in its theoretical and empirical activities and even over the space of a decade it does not seem plausible to claim that nothing socially relevant has taken place. Instead, it is almost certain (and increasingly certain as the pace of change appears to speed up) that some qualitatively new and socially relevant technology or practice like the mobile phone will have impinged on the system over the comparison period^13^.

This situation links to the previous argument about rationality. Conceptually, rational behavior does not cope well with genuine novelty. Do we say that people have always had an implicit “preference” for mobile phones even long before they were conceived of or do we have to explain (using tools that strictly rational approaches generally avoid like learning or imitation) how people come to “acquire” such preferences? By contrast an evolutionary approach says something that is both simple and credible about social responses to novelty. Everyone will approach it with the model of the social world they already have, with the result that some people will adopt it (or at least try it) and others will not^14^. If the technology is incompatible with the worldview of too many people then it will probably “fail.” If it meets a need that people have (even if they don't initially know they have it like the “ego stroking” offered by Facebook “likes”) then it will probably succeed. Evolutionary approaches are thus compatible with genuine novelty in a way that rational approaches are not because they do not require anyone to have views about (let alone be “right” about) things that have not happened yet. All they require is that social actors have a way of dealing with the world as it currently is which can be pressed into service (also with potentially unintended consequences) when new things happen^15^.

It is clear that, although it has relevance to that argument, this is not a grand claim about the role of history (particularly very long term history) in sociology^16^. It is a relatively small-scale empirically based claim about the extent to which genuine novelty and change may undermine our ability to use existing research methods in convincing ways. Thus, as with “perfect models of the world,” sociology faces an unpalatable choice between claiming that there is no genuine novelty in the social world (except perhaps over much longer time scales than apply to sociological research) or having to find new tools to understand its implications for social regularities (existing tools like regression and rational choice theory being problematic in this regard for reasons given above).

Having shown how both theoretical and empirical considerations bear on the need for evolutionary sociology, it is now possible to introduce the third ingredient of the argument. Quite apart from ideological and emotional opposition to evolutionary approaches, there is also (as has been sketched above) a potential limitation of vision imposed by existing research methods. A new tool may help us productively to resolve these challenges in a new way.

## ABM and Evolutionary Analysis: A Case Study

As explained and illustrated in much more detail elsewhere (Chattoe-Brown, 2013), a particular kind of computer simulation (with a distinctive methodology) called ABM is markedly effective at exploring systems of autonomous social actors whose actions may have unintended consequences at the aggregate level which, in turn, may differentially affect the survival of agents carrying out those actions (such as firms in a market or foragers in an ecology). The model presented here is not intended to be realistic at this stage (though a well-established methodology exists to make it realistic later) but to demonstrate how the approach can explore systems in which “genetic” evolution, “social” evolution and adaptive (perhaps ultimately rational) behavior can interact under conditions of macrostructural change and genuine novelty. The behavior is the simplest possible that can nonetheless illustrate the point, namely foraging for food. The simulated world is made up of square patches each of which contains a number of units of food at any point. Normally, the food grows back at one unit per time period until it reaches a maximum and is depleted by consumption from agents on the patch (according to a process that will be described shortly). However, if all the food on a patch is eaten in a single period then the patch becomes “denuded” and food no longer grows back until, with a very small probability, the patch manages to recover at some later stage. This means that if the agents are collectively “too greedy” they will cause environmental collapse. The other simplified element of the environment is a stylized representation of “predators.” These are a small number of patches that, if an agent lands on it, will kill that agent. These patches (which could equally well be viewed as “natural hazards”) are introduced as a contrivance purely to stabilize the population (in the way that real predators/hazards do). A population of unregulated agents without predation will initially boom but then completely collapse. However, as the population increases, the predators will have more prey and this chokes off population growth so that the agents are restrained from invariably destroying the environment (and thus suffering extinction)^17^.

## The Baseline Model: Genetic Evolution Only

In this version of the model, each agent has a “gene” representing the fraction of food that it will forage from a patch. This gene can thus have values between 0 and 1 (in increments of 0.1). An agent with a foraging propensity of 0 will clearly starve to death (as it takes no food) while one with a foraging propensity of 1 will take all the food available (but thus denude the patch). For reasons that will be explained shortly, agents have a “storage capacity” which determines the maximum amount of food they can take with them. If a patch offers more food than they can carry, then they will eat the surplus (and thus gain energy). Living and reproduction both take energy, with the latter taking far more than the former. Agents will reproduce a single offspring when their stored energy reaches a certain fraction of their maximum capacity. (This reproductive capacity does not cover the whole lifetime of the agent having, as with humans, a lower *and* upper limit.) Some of that energy will be used up in the birth process and some of it will be transferred to the offspring. Because merely existing also takes energy, agents who are nearly running out of energy will consume some food from their store (as long as they have some). This means that agents can die from four separate causes: Old age (probabilistically above a certain age), exhaustion (when they can't get the energy they need before they have to expend it on living), predation (described above), and birth (if this requires more energy than the agent has available for it). At birth, the value of the foraging fraction of the parent is transmitted to the child with a 20% chance of decreasing by 0.1 and a 20% chance of increasing by the same amount (subject to remaining in the permitted range 0–1). This means that if the environment changes in such a way that the fraction which worked for previous generations no longer works (through environmental degradation for example) then selection pressure always has some “genetic variation” to work on to permit adaptation to new conditions. Because this is baseline model, slightly different questions are of interest in analyzing its behavior. The first “experiment” was simply to run the simulation ten times. Because it contains various stochastic elements (the initial distribution of food and foraging propensities, exactly when agents have the energy to give birth, whether it kills them and so on), it is an open question whether runs differing only in these stochastic elements reach similar end states. Interestingly, the variation between runs is quite small (using a measure of wellbeing discussed below the maximum value is 1.8% above the mean and the minimum value is 1.7% below it) suggesting that we will not need to do enormous numbers of simulations to compare different sets of social processes. The second issue to be investigated was how best to characterize the outcome quality of different runs. This was done in two ways. One was to count all the simulated time periods for each agent when it was not just about to starve to death. This measure combines the number of agents alive in the world at any given time (more agents means more wellbeing using a utilitarian logic) subject to the constraint that those agents are not in a “bad state” (starving due to over population for example). The other was to look at the fraction of agents who died of old age. Because agents cannot reproduce for their entire life, the fact they live to old age again suggests some notion of “quality of life” in the population/environment balance not driven by reproductive considerations alone.

The behavior of the ABM under these conditions was credible but unremarkable. The fraction of “ground cover” (non denuded patches), average agent food storage, patch food level and agent energy level along with population all achieved a steady state (though with some noise around the typical level). An initial population surge meant that there was a significant reduction from initial levels of ground cover and food levels per patch. Genes for low foraging fractions (all except 0) remained in the population but at very low levels for fractions below 0.6. More interestingly the gene for taking all food on a patch neither disappeared nor dominated the population. Instead it was roughly on a par with the proportion of agents with genes for 0.8 and 0.9 foraging proportions. This suggests that the stabilized population has reached a “mixed” equilibrium in which some agents can denude patches but the low population levels (and small ongoing chance of patch recovery) continue to support that behavior (provided it occurs in combination with some agents taking high but still sustainable levels of food).

This baseline model thus serves several functions. First it introduces the basic operation and “domain” of the exemplar model that can then be developed in later variants at lower cognitive cost to the reader. Secondly, it shows exactly how variation, selection and reproduction at the individual level operate within an explicit representation of their aggregate effects on the environment (which “exercises” selection through the growth/denuding behavior of forage and the exogenous action of stylized predators). However simple and arbitrary the model (recalling that it is only meant to be an illustration of the distinctive contribution of ABM could make to evolutionary sociology), it effectively represents an explicit process of social change in which all assumptions are transparent and this process is clearly an evolutionary one, showing as it does processes of variation (the potential differences of the foraging gene between parents and offspring), reproduction (the linkage between the value of the foraging gene and the capacity to generate offspring) and selection (the effect of the current state of the environment on the relative success of different foraging genes). Note, however, that this baseline model does *not* tackle either of the large critiques raised against non-evolutionary sociology (and it is not intended to). Although it can represent the very long term “history” of a social process, there are currently no “social innovations” or “structural changes.” It also does not yet make sense to talk about “unintended consequences” of actions since there are no intended actions but merely genetic dispositions. These challenges to non-evolutionary sociology will be introduced and analyzed in subsequent variants of the model presented below and this is a deliberate strategy to make a novel argument more manageable.

## Introducing Historical Shocks

For the purposes of this model, it is easy to represent a certain kind of novel “structural change” in the environment^18^. After a certain time period (chosen to ensure that the ABM could reach steady state both before and after the shock), instead of patches growing back one food unit per tick, they grow back 0.1 food units per tick (corresponding perhaps to the effects of global warming). The agents have never been exposed to this situation before so they cannot have adapted to it. It thus represents for them a complete novelty. The results of this set of simulations are somewhat more interesting. As before, the system behaves quite similarly in each simulation run despite the considerable stochastic elements. In three runs out of ten, the shock renders the population extinct (thus suggesting that ABM can cast interesting light on qualitatively different system outcomes and their frequency). In the remaining simulations, the behavior was again quite closely similar between runs. Unsurprisingly there was a sharp drop in population when forage regrowth slowed dramatically. Much more interestingly, the population then recovered to a new steady state not massively below that prior to the shock. However, notably, the population fluctuated much less and there was an intriguing shift in the nature of the environment. Ground cover became almost universal again during the population slump but the average level of forage on each patch obviously dropped dramatically. Thus the fall in food recovery had the apparently paradoxical effect (when coupled with the population drop) of *increasing* ground cover^19^. The other notable effect was that foragers taking all the forage on a patch became dominant at the expense of those taking 0.8 (with those taking 0.9 remaining more or less constant before and after the shock). This shift makes sense in the context of the baseline model however. The environment can support some proportion of agents so greedy that they denude and these will have something of a reproductive advantage. However, these agents cannot become dominant without destroying the ecosystem and this mitigates their reproductive advantage relative to more restrained agents resulting in a mixed equilibrium.

Again, this model variant only takes us a little closer to the full argument which opens the article. The ABM is shown to represent a genuine novelty or structural shift (something no agent has encountered before, occurring at a particular point in time) but because foraging is still based on “genetic disposition” the development of the model does not yet address the “unintended consequences” aspect of the model which will be dealt with shortly in another model variant.

## Social Evolution: Sharing Behavior

One interesting sidelight on evolution is to note that in some sense genetic evolution and certain forms of social evolution appear to be equivalent. To the extent that cultural practices (like genes) remain unchanged for the lifetime of the agent and are only potentially modified during “reproduction” (whether actual gestation and birth or socialization) it is not clear how one has advantages over the other in adapting to the environment^20^. For this reason, the exemplar model presented here does not analyse a social disposition to take food distinct from the genetic one. Instead, the socially evolved behavior analyzed to illustrate the arguments of the article is that an agent with a reasonable store of food can *share* with an agent who is close to exhaustion. As with the previous two experiments, behavior is strongly consistent across runs suggesting that specified processes rather than stochastic elements are dominant in driving the system. To make the point about modeling genuine novelty in a different way, at the start of the simulation nobody shares. Then, with a probability, if there aren't already sharers in the population, this social practice can “dawn on” one agent. This means that to spread to some reasonable proportion of the population, sharing cannot be too genetically disadvantageous. (Note that these agents share with anyone in need and not just kin so this practice does not simply operate as genetic group selection). It turns out, however, that even though roughly half the population ends up willing to share (suggesting that this is a genetically viable social innovation), the actual number of sharing instances is quite small (600 or so over 20,000 simulated time periods). This is partly because of the low population density that can survive with relatively greedy agents, partly because even if an agent is willing to share, they may not have the resources and partly because recipients only qualify when they are very close to exhaustion (and thus only have a narrow window in which help can arrive before they do actually die). As a result, in a world predominantly based on a genetic propensity to forage, sharing has little ability to demonstrate a significant social benefit^21^. (The difference between the means of wellbeing measures in the two conditions is only 0.1%, which is dwarfed by the variation between runs in each condition small though that is^22^).

It is important not to misunderstand the intention of introducing this variant at this stage. It is still merely a “disposition” (albeit social rather than genetic) so the issue of “unintended consequences” is still not yet addressed. However, this variant shows that the ABM is not limited to representing environmental novelty but can also represent *social* novelty and that, furthermore, it can represent a process where social innovations can operate “on top of” the baseline genetic selection process. Here, for reasons based on contingent details of the model, the innovation of sharing does not have a large effect. But it does spread through the population (showing that it does not undermine the population survival created by genetic selection) and does display *some* positive effect as well. Thus we can see how certain sorts of social innovation (again operating via reproduction, variation and selection) can structurally change the nature of the environment over long time scales and be effectively represented by an ABM.

## Adaptive Behavior

We can now add the final component to the model that completes the opening argument: The capability of agents to act in response to the environment *within* their lifetimes and thus create “unintended consequences” through holding particular models of the world^23^. We assume (arbitrarily for illustration) that agents do this by keeping a running total of agents who have been born and died in their vicinity over a certain number of time periods (they “forget” older information as they add newer thus adapting with some inertia but in proportion to the rate of change). If this total is significantly skewed toward an excess of births, then the agent cuts back its foraging fraction by 0.1. If it is skewed toward an excess of deaths, then the agent increases its foraging fraction by the same amount. The agent logic here is that if “too many” agents are dying, more birth (and thus more energy) are needed but if too many agents are already being born then “restraint” is important to avoid environmental degradation. Thus the agent is given a “reasonable” decision making process about the world and the connection between its behavior and potential outcomes desirable for survival^24^. The behavior of this system is considerably and interestingly different from the system driven by genetics with or without shocks and a social disposition for sharing. The most noticeable difference is that exactly the same environment will support an order of magnitude more agents (a couple of thousand rather than a hundred or so)^25^. In the main, the system is stable so this population really is being supported over the long term (20,000 time periods assumed very roughly to be years in terms of things like simulated birth rates and life expectancy)^26^. Another interesting difference is the very high fraction of agents who are “socially adapted” to taking no food from each patch (around 80% of the population). This result needs to be correctly understood because it is no longer a disposition fixed over the life time. Foraging proportions determined genetically would make this strategy clearly unsustainable because such agents could never survive (let alone breed and transmit the disposition). However, under adaptation, it is possible for agents to do this at some times and not others (for the “collective good”) without completely compromising their ability to reproduce (particularly if others are exercising similar restraint). The very high population supported suggests the “wisdom” of this adaptive process (as does its dominance in the population^27^). The results of the wellbeing measures for the system are slightly less clear-cut however. Not surprisingly, with ten times as many agents, welfare measured by the total count of time periods with agents not at “death's door” is nearly ten times greater but the proportion of agents managing to die of old age drops noticeably (and outside the variation range resulting from stochastic effects between runs) from 0.65 to 0.60. Thus, depending on ones conception of the “good life” this system is probably a social improvement but not a completely unqualified one.

The argument of the article, as illustrated by a sequence of variant ABM and their analysis, is now complete. The opening argument suggested that sociologists who wish to remain non evolutionary face two unpalatable choices^28^. Firstly, they either have to maintain the possibility of perfect models of the world or struggle to use existing theories and methods to analyse the inevitable unintended selective consequences of *imperfect* models. Secondly, they either have to maintain (implausibly) the insignificance of structural change (including genuine novelty) or, again, struggle to represent this phenomenon with existing tools apparently unsuitable for the purpose. The models presented show the way out of this impasse. A new method (ABM) is shown to explicitly represent structural change, social innovation, models of the world with unintended consequences and so on and shows how these different phenomena change the dynamics of the system in important ways. Sociologists can, of course, still reject these approaches but this article has both shown the implausibility of the position required to do so (no fallibility, no history) and that a perfectly serviceable method now exists to represent these phenomena for further study (so we are not “obliged” to make these assumptions because of the limitations of existing research methods).

## Discussion

It is very important to be clear what this analysis does (and more importantly doesn't) do. This is clearly not an empirical set of claims about how food collection happens or about what the key determinants of social survival are. However, it does demonstrate how an ABM allows us to represent systems in which there may be genetic and social/cultural selection processes (fixed over the life of the individual) as well as adaptive behavior (changing over the life time of the individual in response to the environment and other agents). It also allows us to see how different adaptation mechanisms respond to an environment operating according to exogenous processes (like plant behavior, predation or a “regrowth shock”) and how the collective effects of individual actions (or choices) may be unintended and counter-intuitive (like the shift from low ground cover with high food levels to high ground cover with low food levels under a “regrowth shock”). However, such a model goes further in structuring ideas about how we would get the data we needed to make the model more realistic and why existing forms of data might need to be treated with caution. Some data, like the actual cultural norms regarding sharing of food (who with, when and how much) are exactly what ethnography elicits (for traditional societies at least). “Simple” correlations (like the probability that parents and children will have the same attitude to sharing) are also routinely analyzed statistically in sociology (for example in modeling social mobility or voting behavior). The slightly trickier kind of data, which is currently at the frontiers of the social sciences, involves any attempt to characterize relationships between genetic phenomena and social behavior (Harrati, 2014). Such approaches are not necessarily intrinsically difficult to implement (though they do rely on genetic information that has only recently become reliably available in quantity) but they do raise important issues of research design (any such correlation we discover is mediated by large quantities of socialization and social interaction between birth and sampling and should therefore be interpreted with caution) and academic culture: Sociologists are not at all comfortable with the idea of “admitting” the ongoing role of genetics in human behavior in case it becomes a rationalization (probably spurious) for biological reductionism or inhumane kinds of social intervention. ABM also makes it much easier to see why existing correlational approaches might be problematic. At any moment in time, it will be possible to derive a correlation between, for example, the fraction of agents in the population sharing and the level of ground coverage. However, it is now clear why such a correlation is not causal in any useful way and why it is highly unlikely to remain stable. The actual process linking sharing and ground cover can be seen to include many more “elements” that the correlation credits and, furthermore, this process is not “reducible” to the additive effects of a set of linear elements (Abbott, 1988) so this isn't just a matter of not putting enough variables in the regression but a fundamental mismatch between what statistical models are equipped to represent and how we have reason to think the world works when we consider it from this novel perspective^29^.

Even with a “toy” ABM (that only has the broadest correspondence to reality) further explorations with the model could usefully be carried out (though not realistically within the constraints of a single article). While exploring alternative assumptions will not tell us what is “true” (only real data and systematic methodology will do that) it would give a better sense of which outcomes are most heavily contingent on particular assumptions. For example, how much does it matter to birth rates if agents immediately eat any food that they cannot carry but are “genetically compelled” to forage. What would happen to the system if they left the surplus behind instead? Similarly, the adaptation mechanism proposed here is quite altruistic and backward looking (involving births and deaths in the *population* rather than the situation of the agent itself). What effects would other kinds of adaptation mechanism have? For example, what if agents monitored the average level of ground cover or food available and adapted their behavior to that or, rather than only adapting based on past data, tried to refine a model that made predictions about *future* ground cover and available food levels and was judged on the success of those predictions (probably the nearest one can get to “rationality” in a complex system with imperfect information). Finally, does the nature and frequency of shocks have any bearing on the relative merits of genetic and social evolution relative to individual adaptation and if so, how can that be explained? (So far I have only examined the effect of a single shock in each simulation run.) It seems intuitive that relatively stable environments may be better handled by slower adaptation schemes (genetic and social evolution) but this intuition may be undermined by the interaction of different processes^30^. However, ABM gives us a unique tool for representing (and thus gaining better understanding of) such variant systems and their behaviors. At the same time, however, such speculative analysis should not persist indefinitely. There does need to come a point at which we seek to establish which variant model best corresponds to reality and best tracks real data. [The methodology for this kind of analysis and its relationship to data is already well known in ABM and is discussed, for example, by (Gilbert and Troitzsch, 2005)].

## Conclusions

It is always hard to argue convincingly about why things *don't* occur but I have tried hard to suggest in this article why evolutionary analysis may be subjected to a “triple whammy” relative to sociological practice. In the first place, sociologists don't much seem to “like” this approach for a variety of reasons (concern with biological reductionism, the perception that functionalism is fully discredited along with the peripheral status of its successors, the possibility of illiberal views of society based on genetic arguments and so on). In the second, sociology is notoriously eclectic in its approach and that makes it difficult to establish a disciplinary core determining what kinds of analysis sociologists need to be able to do. Historical sociologists (who are largely not quantitative) form one community. Social statisticians, who often use data collected at different times but do not seem to worry about the consequent role of historical change, form another. There doesn't seem to be an existing approach that can reconcile those differing positions or a sense that it might need to be done. This is the third part of the triple whammy, that evolutionary analysis involves a different conception of how systems work (process based rather than number or narrative based) and to even begin to talk about this we need a different approach to theorizing/modeling than those currently widely accepted in sociology. I have attempted to show how ABM can support this approach while still not divorcing itself too radically from existing sources of data and research methods (though these methods and data may have to be *reoriented* somewhat to the new approach).

The argument now returns to its origins at the beginning of the article with the additional weight of the model analysis behind it. If we don't want to allow for any possibility of selection by the social environment (which is obviously not the same as saying that selection is the only force in operation—the boundary between rationality and selection is a fascinating and under explored area), then we are obliged to assume that agents have “perfect” models of the world which are regarded as conceptually problematic even in the few areas, like Rational Choice Theory, where they are countenanced (Richter and Wong, 1999). Even if we can stomach this position we are also obliged to claim that the social world experiences no change or, if it does, that this change is of a kind that has no bearing on the “feasibility” of the numbers or narratives which sociologists currently analyse to explain it. (I have suggested—and the model illustrates—why this may not be a convincing position.) By contrast, the ABM presented here shows exactly the sorts of things that can happen when agents have *imperfect* models of the world and when “structural” change occurs and how we can analyse the results. It is possible to believe in a world of perfect models and “unchallenging” change (although easier if existing research methods do not draw attention to the implicit difficulties) but given what sociologists currently study it is not *easy* to do so. In particular, while historical sociology over centuries or millennia may be hived off and disregarded as a different area with different rules, combinations of social evolution and adaptation to structural change can importantly affect the social system over mere decades or perhaps even shorter periods (and existing sociological research, on social mobility for example, easily operates within this time scale and is therefore subject to the potential challenge of evolutionary analysis.) I have tried to show in this article both how it is empirically and conceptually hard to believe what is necessary to do without any evolutionary analysis in sociology and also how a novel research method (ABM) makes maintaining such awkward beliefs completely unnecessary.

## Author Contributions

The author confirms being the sole contributor of this work and has approved it for publication.

### Conflict of Interest Statement

The author declares that the research was conducted in the absence of any commercial or financial relationships that could be construed as a potential conflict of interest.

## Footnotes

^1^This inability to “kill off” evolutionary thinking, despite the number of times it has not just been supposedly discredited but actually reviled, is itself potentially interesting. In addition, these and related ideas are found in social science outside sociology, for example in management (Aldrich and Ruef, 2006; Aldrich, 2008).

^2^Notice that I have been careful to talk only of social actors. These could be individuals attempting to maximize their wellbeing in rational terms or they could be firms trying to agree on a pricing strategy. The core argument about the quality of “models” and the likelihood of unintended consequences remains though the details of what the models are, how they are formulated and evaluated will certainly vary in different social contexts.

^3^Again, models of learning are an obvious adjunct to understanding this process though fairly uncommon in sociology [but see, for example, (Macy, 1991)]

^4^In other words, there is no “invisible hand” as economics sometimes postulates.

^5^Simple economic theory also assumes that *consumers* are perfectly informed and do not incur (for example) travel costs. This means (at least in popular accounts of perfect competition) that if one firm is charging a penny less than any other, it will scoop the whole demand. In the real world, we would expect travel costs, brand loyalty, non-price features, lack of information, and a whole raft of other things to mean that there was some “stickiness” in the process by which customers change who they buy from. Nonetheless, in the longer term we would still expect a firm charging a high price to lose out to one charging a low price.

^6^It is this fact that underpins the extensive data sets that are analyzed by organizational ecologists (Hannan and Freeman, 1988).

^7^This should not be seen as an argument against functionalism or its subsequent developments. No theory of social action can credibly be applied generically. It is a matter of sociological skill to draw on theories appropriately for particular social phenomena.

^8^The predominance of *narrative* theories, which may be hard to formulate or critique definitively, may also contribute to the contentious nature of evolutionary theorizing in sociology to date.

^9^This is a simplification that can be well understood by a concept from evolutionary game theory (Maynard Smith, 1982). Early in the history of a particular industry a larger firm may be able to drive out smaller ones. However, “the industry” is not an unchanging thing and it does not therefore follow that small firms can never survive in that market. What we are really talking about as regards the evolutionary process in a market are firms that can persist against diverse changes in the firm population (what is called a non-invadable strategy in evolutionary game theory). Of course no firm or strategy can survive against all possible situations (like the total collapse of demand or competition to the death from a large firm previously outside the industry). Robustness (as in biological evolution) is necessarily a relative and not an absolute term.

^10^For reasons of space, this article does not attempt to model institutional evolution as well but we have attempted a very stylised version of this in a previous publication (Chattoe-Brown and Gabbriellini, 2016).

^11^Interestingly, this is an area where qualitative and quantitative research methods prove to be rather alike. Exactly the same problem faces the interpretation of two sets of qualitative interviews conducted a decade apart on the “same sampling population” whether they prove to be rather similar or rather different.

^12^This is the point at which we can no longer be agnostic about the virtues of regression. For the parameters to remain stable, one might have to postulate some sort of social regulation mechanism that prevented other changes invariably occurring in the system from altering this relationship. But the regression analysis would be completely incapable of identifying (let alone confirming or refuting) the existence of such a mechanism.

^13^There are, of course, longitudinal statistical techniques but they are very rare in sociology and face challenges of their own as regards historical change. For example, can a major structural change be effectively represented as a binary dummy variable? (War World II is occurring/World War II is not occurring.) There are also various simulation and equation based approaches which might address the problems I raise but I have not seen them used in the context of evolutionary sociology. The case I am making here is not intended to rule out other approaches however.

^14^This also resolves a potential inconsistency in certain models of innovation diffusion. Any assumptions about learning, imitation and so on have to involve a coherent explanation about why some people *don't* have to learn, imitate and so on. Logically not everyone can be a follower!

^15^This issue draws attention to the possibility of other decision processes that are plausible but neglected in social science such as Case-Based Reasoning (Gilboa and Schmeidler, 1995). In CBR, the agent matches the new situation to the “closest” situation they already know about and decides accordingly. This approach has other appealing features. For example, an “expert” is someone who has a lot more situational matches based on experience and/or is better at matching new situations to existing ones. This means that their judgments are more likely to be effective than those of novices (who may have to match a situation that is very distant from any of their experience or may match ineptly through inexperience). Such an approach thus links the nature of experience and the development of expertise in a credible way.

^16^While I personally find Runciman's arguments about selectionism careful and thought provoking, their wider acceptance may be limited by his very sociologically atypical application of these ideas to very long term historical change. I really cannot decide based on his writings whether his approach, applied to the growth of Christianity (Runciman, 2004) over millennia could also legitimately be applied to changes of the UK education system after the creation of the welfare state. It seems to me (for reasons given in this article) that the need for evolutionary accounts still exists over recent decades (which are short enough periods not to be “purely” historical but long enough to make the typical sociological assumption that structural change or genuine novelty are irrelevant rather risky) and not merely over centuries.

^17^Because this model is so stylized, it is not helpful to report quantitative information about parameter values and results (since all that would do is give a spurious sense of precision). All the relevant materials (code, data files and images of simulation runs) are, however, available on request from the author.

^18^Recall that this is only an example so just a single kind is illustrated. Many other kinds are possible like the development of new artifacts, new social arrangements and so on. Analysis of these must be left to subsequent research.

^19^A referee has drawn my attention to the fact that analogous effects are already known in ecology as the “paradox of enrichment” (Rosenzweig, 1971) and the “hydra effect” (Abrams, 2009). This perhaps suggests some very weak face validity in the assumptions of my model.

^20^In fact, this may reflect an overly naïve view of the transmission of cultural practices (and particularly socialization) in the simple model. For example, such practices may have to be enforced by social norms (which may themselves shift) or parents may, even though they were socialized in a certain way, observe that the world has changed and refuse to enforce that socialization so strongly on their offspring. This is an area that would benefit from further research and conceptual development particularly given the wider recent neglect of socialization in sociology.

^21^In a world containing a shock there is even less sharing suggesting that although this behavior has the potential to be helpful in a crisis, in practice there are not the resources available to share.

^22^As a very informal confirmation of this, a simulation run combining sharing and adaptive behavior (which results in much higher levels of population as discussed) also produced much higher levels of sharing for the same sharing process. Interestingly this variant also displayed several “endogenous slumps” toward population collapse although in each case the agent population recovered.

^23^Note that I have followed the logic of the opening argument here. It is difficult to model “rational” behavior in this context because it is not clear what that would consist of. However, adaptation in the way specified is “reasonable” behavior (thus serving as a fair illustration of the opening argument by leading to a potential for “unintended consequences.”) Adaptation to the current environment based on a “sensible” response (be less greedy when more agents are being born around you needing to be fed) can no longer be viewed merely as a disposition.

^24^In fact, of course, part of the point is that such a simple model is very prone to unintended consequences because it does not incorporate such important aspects as partial knowledge of the environment, the “non-negotiability” of genetic dispositions and so on. However, far from making the model merely “unrealistic”, this fits it particularly well to illustrate the point of the article. Sociology is *still* not good at conceptualizing the limitations on social organization imposed by genetic dispositions!

^25^For this reason, I have only reported the value of a single run here since they are much slower. However, I have no reason to think from the analysis I did previously (and reported here) that the outcomes of individual runs vary hugely from stochastic effects.

^26^Having said this, however, the system displays one interesting result not specifically for the analysis but for the behavior of complex systems in general. “For no reason” after about 4,000 time periods there is a sharp dip in population mirrored by the fraction of ground cover but not in the average forage level or the energy and stored food of agents). The system recovers to its previous typical values but this “endogenous spike” is another feature of complex processes (resulting perhaps from a rare “coincidence” of stochastic effects).

^27^For reasons of space it has not proved possible to consider all possible variants of selection and adaptive behavior and I have focused on the simplest examples that illustrate my core argument. Another obvious mechanism involves so called evolutionary psychology (Buss, 1995) in which brain structures and resulting capabilities (like altruism) may be genetically selected and create social advantage. Although adaptive behavior diffuses through the population in my last variant model I have not been specific about whether this is a social innovation or a “genetically transmitted disposition” because for the purposes of the argument (which is to study the *effect* of “imperfect models” rather than their origins) it doesn't matter.

^28^They may of course face more than two challenges but these two alone seem quite sufficiently “awkward” to justify attempting a new approach.

^29^If you only have statistical methods, and these are clearly more “rigorous” than casual analysis, then the temptation is to presume that it is reasonable that the approach should work (and there is nothing in the outcomes of the analysis to suggest that it actually doesn't). At worst you get a poor correlation and assume that this means you haven't just found the right variables yet or that the social world is just generally “tricky to explain”. But, of course, you will also get a poor correlation if the world is complex (which means that assumptions of normality and linearity on which statistical models are built do not in fact hold). It then becomes a matter of faith how the difficulty will ultimately be fixed and it is easier to maintain this faith if one does not consider the implications of ABM and complexity as sociology generally does not

^30^There are subtleties here. Strictly this ABM does not “combine” genetic and adaptive behavior because foraging is driven by one or the other. This raises the question of how, in fact, any genetic “dispositions” humans have can be regulated socially. If ones “inclination” is to take all the food on a patch, can social convention effectively ensure that you take *none* of it or is the best that can be achieved that you take “somewhat less” than you are inclined. As far as I know, the answers to such questions are open in sociology because sociologists seem disinclined to ask them. Nonetheless, any genetic limitations on the mutability of human behavior would seem to have extremely important implications for social policy. Do we eat so much sugar we give ourselves diabetes merely because we are contingently greedy? Will a “sugar tax” therefore deal effectively with our greed?
